# A1899, PK‐THPP, ML365, and Doxapram inhibit endogenous TASK channels and excite calcium signaling in carotid body type‐1 cells

**DOI:** 10.14814/phy2.13876

**Published:** 2018-10-04

**Authors:** Peadar B. O'Donohoe, Nicky Huskens, Philip J. Turner, Jaideep J. Pandit, Keith J. Buckler

**Affiliations:** ^1^ Department of Physiology, Anatomy and Genetics University of Oxford Oxford United Kingdom; ^2^ Nuffield Department of Anaesthetics Oxford University Hospitals Oxford United Kingdom

**Keywords:** Carotid body, Hypoxia, Kcnk3, Kcnk9, Potassium Channels, TASK

## Abstract

Sensing of hypoxia and acidosis in arterial chemoreceptors is thought to be mediated through the inhibition of TASK and possibly other (e.g., BK_C__a_) potassium channels which leads to membrane depolarization, voltage‐gated Ca‐entry, and neurosecretion. Here, we investigate the effects of pharmacological inhibitors on TASK channel activity and [Ca^2+^]_i_‐signaling in isolated neonatal rat type‐1 cells. PK‐THPP inhibited TASK channel activity in cell attached patches by up to 90% (at 400 nmol/L). A1899 inhibited TASK channel activity by 35% at 400 nmol/L. PK‐THPP, A1899 and Ml 365 all evoked a rapid increase in type‐1 cell [Ca^2+^]_i_. These [Ca^2+^]_i_ responses were abolished in Ca^2+^‐free solution and greatly attenuated by Ni^2+^ (2 mM) suggesting that depolarization and voltage‐gated Ca^2+^‐entry mediated the rise in [Ca^2+^]_i._ Doxapram (50 *μ*mol/L), a respiratory stimulant, also inhibited type‐1 cell TASK channel activity and increased [Ca^2+^]_i._. We also tested the effects of combined inhibition of BK_C__a_ and TASK channels. TEA (5 mmol/L) slightly increased [Ca^2+^]_i_ in the presence of PK‐THPP and A1899. Paxilline (300 nM) and iberiotoxin (50 nmol/L) also slightly increased [Ca^2+^]_i_ in the presence of A1899 but not in the presence of PK‐THPP. In general [Ca^2+^]_i_ responses to TASK inhibitors, alone or in combination with BK_C__a_ inhibitors, were smaller than the [Ca^2+^]_i_ responses evoked by hypoxia. These data confirm that TASK channel inhibition is capable of evoking membrane depolarization and robust voltage‐gated Ca^2+^‐entry but suggest that this, even with concomitant inhibition of BK_C__a_ channels, may be insufficient to account fully for the [Ca^2+^]_i_‐response to hypoxia.

## Introduction

Peripheral chemoreceptors provide vital information regarding blood gas and pH levels to both respiratory and cardiovascular control centers (de Burgh Daly 1997). The best understood of these organs is the carotid body. This chemoreceptor is able to sense changes in blood oxygen, CO_2_, and pH within seconds. The carotid body contains specialized sensory cells called type‐1 or glomus cells which respond to hypoxia, hypercapnia or acidosis by generating a depolarizing receptor potential which initiates electrical activity, voltage‐gated calcium entry, and neurosecretion (Buckler 2007; Nurse 2010). This causes excitation of afferent nerves which project to the respiratory and cardiovascular control centers in the brainstem.

How these electrical events in the type‐1 cell are brought about is one of the most fundamental questions in the field. While many ion channels will undoubtedly be involved in shaping the electrical properties of these cells there is intense interest in the nature and roles of those channels that seem to be directly modulated by hypoxia and acidosis. To date, relatively few ion channels have been identified as being oxygen or acid sensitive in these cells. One of these, a background potassium current, is inhibited by both hypoxia and acidosis. This event directly contributes to the initial depolarization (Buckler and Vaughan‐Jones 1994a; Buckler 1997; Buckler et al. 2000). This background K‐current is thought to be mediated by members of the TASK channel family predominantly TASK‐1/TASK‐3 heterodimers with minor contributions from homodimeric TASK‐1 and TASK‐3 channels (Kim et al. 2009; Turner and Buckler 2013). These channels will henceforth be referred to collectively as TASK channels. Other hypoxia and acid sensitive currents have also been described in type‐1 cells including a delayed rectifier K –channel (Lopez Barneo et al. 1988; Lopez Lopez et al. 1989; Ganfornina and Lopez Barneo 1992) in rabbit type‐1 cells and a large conductance Ca‐activated potassium channel (BK_Ca_) in rat type‐1 cells (Peers 1990a; Wyatt and Peers 1995). The role of these channels is less clear cut with some authors suggesting that inhibition of maxi‐K channels may contribute to initial membrane depolarization whereas others find that inhibitors of these channels often have no effect upon resting membrane potential, or [Ca^2+^]_i_ (Buckler 1997; Pardal et al. 2000; Dallas et al. 2009; Wang and Kim 2017). An alternative perspective is that these channels may act as a brake on cellular excitability for example by limiting membrane depolarization and/or action potential frequency/duration, which can be released in the presence of chemostimuli (Gomez‐Nino et al. 2009).

Our current basic model of chemotransduction is therefore as follows (1) TASK channel inhibition by hypoxia/acidosis initiates depolarization; (2) depolarization activates voltage‐gated calcium channels; (3) rise in [Ca^2+^]_i_ activates a calcium‐dependent cation current which reinforces membrane depolarization; (4) depolarization and rise in [Ca^2+^]_i_ leads to activation of Kv and BK_Ca_ channels which oppose depolarization/electrical activity; (5) Kv and/or BK_Ca_ channel activity is moderated by hypoxia/acidosis (depending on species)(Buckler 2015). Thus, chemostimuli may be considered to exert control over type‐1 cell excitability and calcium signaling at two points – initiation of excitation and a negative feed‐back loop limiting excitation. This might be considered equivalent to controlling the threshold and gain of this sensor.

The objectives of this study were first to confirm the identity of the background channels using selective inhibitors. Second, to establish whether pharmacological inhibition of TASK can increase [Ca^2+^]_i_. The above model of cell excitation in hypoxia predicts that this be true. Third, to determine whether responses to selective TASK channel inhibition can be enhanced by concomitant inhibition of BK_Ca_ (and Kv) channels (another prediction of the above model of cell excitation by hypoxia). And finally to determine whether concomitant inhibition of both TASK and BK_Ca_ can replicate the effects of hypoxia, that is, is the above model sufficient to explain the excitatory response to chemostimuli.

This study successfully confirmed the identity of the background K‐channels as being TASK channels. We demonstrate that pharmacological inhibition of TASK (A1899, PK‐THPP, ML365, and Doxapram) does indeed lead to voltage‐gated calcium entry and increase in [Ca^2+^]_i_. These data confirm the pivotal role that TASK channels play in peripheral chemoreceptor excitation. Concomitant inhibition of BK_Ca_ at the same time as TASK inhibition slightly augmented [Ca^2+^]_i_ supporting the idea that BK_Ca_ inhibition can in principle enhance excitatory responses initiated by TASK channel inhibition but suggesting that this effect may be rather minor. We also observed that TASK channel inhibition with or without concomitant inhibition of BK_Ca_ cannot fully replicate the effects of hypoxia. Collectively these data support key elements of the model outlined above but suggest that it is as yet incomplete.

## Methods

### Carotid body type‐1 cell isolation

Carotid artery bifurcations were dissected from neonatal (P11‐21) Sprague‐Dawley rats (Harlan, Blackthorn, Oxfordshire, UK) under terminal isoflurane anesthesia (4% in oxygen) and placed in ice‐cold phosphate‐buffered saline. Animals were then killed by decapitation or exsanguination while still deeply anesthetized. This procedure was performed in accordance with project and personal licence authorities issued under the UK Animals (Scientific Procedures) Act, 1986. Microdissection of the carotid body was then carried out ex vivo. Carotid bodies were transferred to an enzyme media containing trypsin 0.4 mg/mL (Sigma, Dorset, UK) and collagenase 0.6 mg/mL (Worthington) dissolved in Ham's F12. Tissue was incubated in this media for 25–30 min. The carotid bodies were then washed in culture media comprising either Ham's F12, or a 1:1 mixture of Ham's F12 and DMEM, which were supplemented with 10% fetal bovine serum, glutamine (2 mmol/L), insulin (8 *μ*g/mL), penicillin (100 units/mL), and streptomycin (100 *μ*g/mL) (supplied by Sigma or Thermo‐Fischer scientific). The carotid bodies were then transferred to a small volume of the above culture media supplemented with trypsin inhibitor (0.5 mg/mL: Sigma) and triturated for 3–5 min with fire polished glass pipettes. The resultant cell suspension was then plated out on to 6 mm diameter glass coverslips precoated with poly‐d‐lysine. The cells were left in the incubator to adhere to the coverslips, which were contained within a plastic culture dish, for 2 h before further culture media was added to the dish. Cells were maintained in this media for 0–6 h before further use.

### HEK cell culture and expression of TASK channels

Rat‐TASK‐1 and Rat‐TASK‐3 were cloned into the pIRES‐EGFP plasmid (Clontech/Takara Bio, CA, USA). Human Embryo Kidney cells (HEK‐293; Sigma (Dorset, UK, European Collection of Cell Culture Lot 13F007 P65) were cultured in MEM supplemented with 10% fetal bovine serum (both Gibco, Paisley, UK), 2 mmol/L l‐glutamine (Sigma, Dorset, UK); 100 U/mL penicillin, and 100 *μ*l/mL streptomycin (Fisher Scientific). Cells were passaged every 3 days. On the day prior to transfection ~6 × 10^5^ HEK cells were plated in a well of a six‐well plate with 3 mL which resulted in 70–90% confluency on the day of transfection. Transfection was performed using 800–2500 ng DNA, opti‐MEM, plus‐reagent and lipofectamine (Life Technologies) in accord with the manufacturer's instructions. 24–48 h post transfection, cells were harvested and plated onto poly‐L‐lysine coated glass coverslips and ~2 h after plating, the cells were used for recordings.

### Calcium measurement

Prior to calcium measurements Indo‐1 AM was added to the culture media in the dishes containing isolated type‐1 cells to a final concentration of 2.5 *μ*g/mL. In some instances rhodamine‐conjugated peanut agglutinin 2 *μ*g/mL (Vector laboratories, Burlingame, CA) was also added to this media to facilitate Type‐1 cell identification. Cells were incubated in this media at room temperature and in the presence of 5% CO_2_ for a further hour before use.

Following Indo‐1 loading, cells were transferred to a rapid perfusion bath mounted on the stage of an inverted microscope (Nikon Diaphot, Kingston, Surrey, UK) and observed through a 40x oil‐immersion objective. For this study, we used either isolated single cells or small clusters of 2–5 Type‐1 cells. Indo1 fluorescence was excited at a wavelength of 340 nm by filtered light from a xenon arc lamp. Emitted fluorescence was then measured at 405 nm and 495 nm using a dichroic mirror (450 nm), bandpass filters with a 20 nm bandwidth and cooled trialkali photomultiplier tubes (Thorn EMI, London, England, UK). The output from the photomultipliers was recorded using a CED 1401 and Spike2 software (Cambridge Electronic Design, Cambridge, UK). Signals were averaged over 0.5 sec intervals to give a sampling rate of 2 Hz. Intracellular calcium concentration ([Ca^2+^]i) was estimated from the ratio of recorded fluorescence 405/495 and the equation (Grynkiewicz et al. 1985):

$${\left[{\text{Ca}}^{2+}\right]}_{i}=K_{d}\left(F/B\right)\left(R-R_{\min}\right)/\left(R_{\max}-R\right)$$

Where *R* is the measured ratio, *K*_*d*_ is the dissociation constant for indo‐1 and Ca^2+^ binding (assumed to be 250 nmol/L), *F*/*B* is the ratio of the fluorescence of free and bound forms of indo‐1 at 495 nmol/L, *R*_min_ is the fluorescence 405/495 ratio at nominally zero Ca^2+^ and *R*_max_ the 405/495 ratio of fluorescence at saturating calcium ion concentration. These two latter constants were empirically determined in separate group of cells rendered calcium permeant with 5 *μ*mol/L ionomycin in the presence of 10 mmol/L EGTA in a HEPES‐buffered (pH 7.4) high K+ (140 mmol/L KCl) saline (0 mmol/L Ca^2+^) to determine Rmin and then transferred to 10 mmol/L CaCl_2_ (also in a HEPES‐buffered high K^+^ saline) to determine Rmax.

Unless otherwise stated, calcium measurements are presented either as an absolute mean value of [Ca^2+^]_i_ measured over a >= 40 sec interval or as the difference between two such measurements as a Δ[Ca^2+^]_i_ (e.g., the difference between [Ca^2+^]_i_ measured over a control interval and during the application of a drug).

### Electrophysiology: native glomus cells and HEK293 cells

Cell‐attached patch clamp recordings were performed on glomus cells (isolated as described above), or HEK 293 cells (transfected with TASK1 or TASK3), using an Axopatch 200B (Molecular Devices LLC, Sunnyvale, US). Borosilicate pipettes (Harvard Apparatus Ltd, Kent, UK) were sylgard‐coated, fire‐polished and filled with a solution containing (in mM) 140 KCl, 1 MgCl_2_, 1 EGTA, 10 HEPES, 10 tetraethylammonium (TEA), and 5 4‐aminopyridine. The pH of this solution was adjusted to 7.4 at 37°C using KOH.

Cells were initially placed in the recording chamber and superfused with standard Tyrode. Patching, and seal formation was performed in this solution. Once a satisfactory cell attached patch and gigaohm seal had been formed the standard Tyrode was replaced with a Tyrode solution containing 100 mmol/L K^+^ (in which the Na^+^ concentration had been reduced by 95.5 mmol/L to maintain constant osmolality but was otherwise identical to the standard Tyrode). A pipette potential of +80 mV was then applied and recording of channel activity commenced. Membrane current was recorded at 20 kHz and filtered at 2–5 kHz prior to analysis. Voltage clamp control, data acquisition and analysis were performed using Spike2 (Cambridge Electronic Design, Cambridge, UK). The main conductance state for channel activity was established from an all‐points histogram. Most all‐points histograms showed one main peak at approximately 2.1 nA. The threshold for quantifying nPopen was set at 75% of this value (approximately 1.5 nA), so as to best capture TASK‐1/3 heterodimer channel activity. Current levels that exceeded 175% and 275% of the main conductance state were counted as double or triple openings, respectively.

### Solutions and cell superfusion

Cells transferred to the experimental chamber/bath were initially superfused with warm (36–37°C) Tyrode of the following composition in mM: 117 NaCl, 4.5 KCl, 1 MgCl2, 23 NaHCO3, 11 glucose, this solution was pre‐equilibrated (by bubbling) with 5% CO_2_ in air; pH 7.4. Solutions were delivered to this chamber by gravity from stoppered glass bottles maintained in a water bath at 37°C via two stainless steel perfusion lines and a two way tap placed in close proximity to the chamber. This allowed one perfusion line to run into the chamber and the other to be diverted to waste. Switching between lines could then be effected via the tap. Chamber volume was approx. 100 *μ*l and the perfusion rate approx. 4 mL/min. Temperature was maintained at 36–37°C using an additional heating element either between the tap and the bath or immediately upstream of the tap.

At the beginning of most calcium recordings cells were initially exposed to a hypoxic Tyrode for about 1 min. Hypoxic Tyrode had the same composition as the standard Tyrode but was equilibrated/bubbled with 5% CO_2_/95% N_2_. During a 1‐min exposure to this solution chamber PO_2_ typically reaches a nadir of about 2 mmHg (as measured with a 50 *μ*m optical oxygen sensor: PreSens, Regensburg, Germany). Brief exposure to this stimulus evokes a rapid and robust rise in [Ca^2+^]i (a calcium “transient”) which serves to confirm the identity and oxygen sensitivity of the cell/s about to be studied.

Drugs were added to solution immediately prior to use from concentrated stocks in DMSO or water as appropriate. The drugs used in this study included: A1899 and PK‐THPP (Aberjona Laboratories Boston, MA, USA); ML365 (Tocris); paxilline and iberiotoxin (Cayman Chemicals, Ann Arbor, USA); TEA (Sigma); Doxapram (Abcam, Cambridge, UK).

### Statistical analysis

N values refer to independent recordings from a different cell/s on different coverslips. Generally, where paired data were compared, Student's *t* test was used, unless unequal variance was demonstrated, in which case Wilcoxon Signed‐rank was used. In concentration response curves correlation of concentration and response was analyzed using Spearman's Rho or by a one‐way repeated measures ANOVA. The research materials supporting this publication can be accessed by contacting Dr K. J. Buckler.

## Results

### Confirming action of PK‐THPP and A1899 on TASK‐3 and TASK‐1 channels, respectively

**Figure 1:**
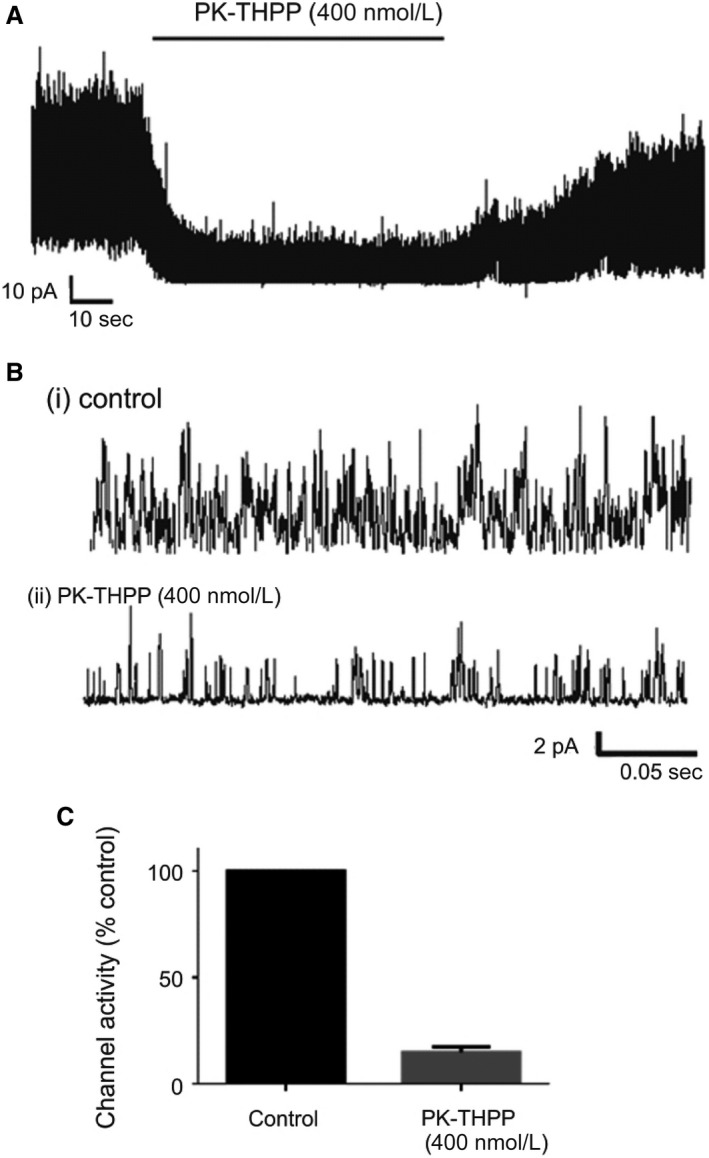
(A) Representative recording of channel activity of TASK‐3, expressed in a HEK 293 cell, over ~2 min in a cell attached patch recording. Note marked inhibition of channel activity by 400 nmol/L PK‐THPP. (B) Data from the same recording over ~0.3 sec under control conditions (i) and in the presence of 400 nmol/L PK‐THPP (ii). (C) Bar chart comparing effects of 400 nmol/L PK‐THPP on single‐channel activity (nPopen) relative to control (*n* = 3, *P* < 0.001). Values are means ± SEM.

**Figure 2:**
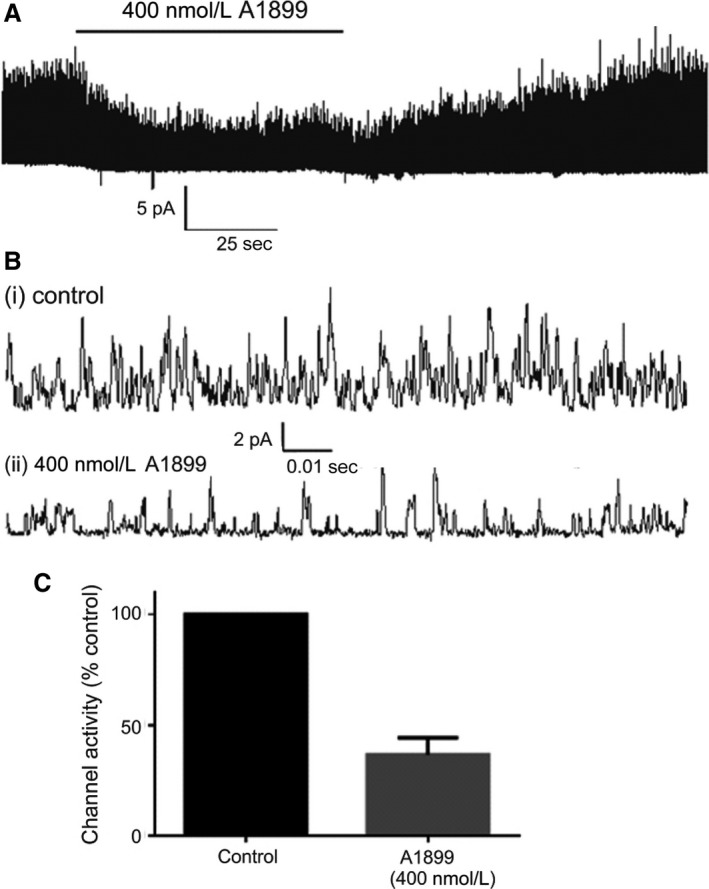
(A) Representative cell‐attached patch recording of channel activity for TASK‐1, expressed in a HEK 293 cell, over a ~3 min period demonstrating inhibition by 400 nmol/L A1899. (B) Data from same recording over ~0.1 sec intervals under control conditions (i) and in the presence of 400 nmol/L A1899 (ii). (C) Bar chart comparing effects of 400 nmol/L A1899 on single‐channel activity (nPopen) relative to control (*n* = 4, *P* = 0.045). Values are means ± SEM.

We first confirmed that PK‐THPP and A1899, reported to be moderately selective inhibitors of TASK‐3 and TASK‐1, respectively (Streit et al. 2011; Coburn et al. 2012; Kiper et al. 2015), did indeed inhibit these channels when expressed in HEK 293 cells and studied using cell attached single‐channel recording techniques, that is, under the same conditions as those to be employed in studying type‐1 cells. Expression of either channel resulted in an abundance of channel activity with multiple channels frequently present in each cell attached patch (see Figs. 1, 2). Upon application of PK‐THPP (400 nmol/L), to TASK‐3 expressing cells, or A1899 (400 nmol/L) to TASK‐1 expressing cells there was a marked reduction in channel activity with residual channel openings becoming more clearly resolved (Figs. 1, 2). PK‐THPP inhibited TASK‐3 channel activity by 85.1 ± 2.6% (*n* = 3; *P* < 0.001). A1899 inhibited TASK‐1 channel activity by 63.4 ± 7.7% (*n* = 4, *P* = 0.045).

### Effect of PK‐THPP and A1899 on TASK channel activity in native glomus cells

#### PK‐THPP

**Figure 3:**
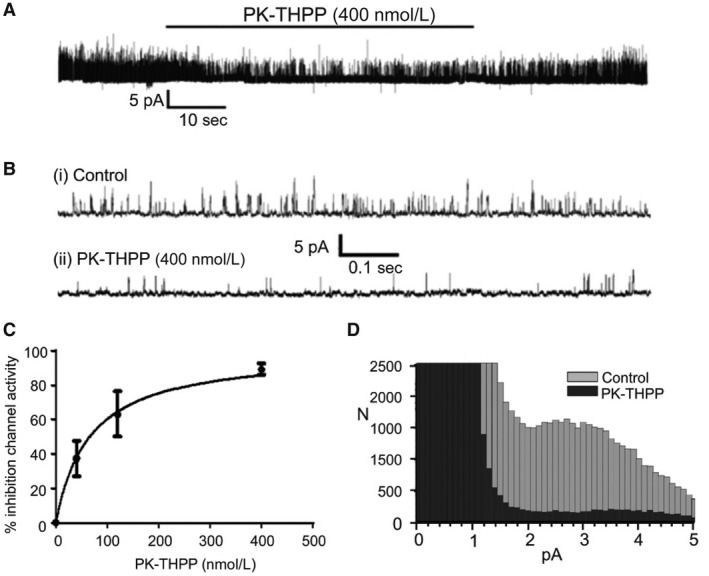
(A) Representative recording of type‐1 cell TASK channel activity over ~1.5 min in a cell‐attached patch showing inhibition of channel activity by 400 nmol/L PK‐THPP. (B) Data from same recording over ~1 sec intervals under control conditions (i) and in the presence of PK‐THPP. (C) Concentration‐response relationship of effects of PK‐THPP on rat type‐1 cell TASK channel activity (nPopen values in the presence of PK‐THPP expressed as % inhibition of control nPopen values). Data are mean ± SEM;* n* = 5 for 40 nm and 120 nmol/L PK‐THPP and *n* = 10 for 400 nmol/L PK‐THPP. Data were fit to a single‐site ligand binding model with *r* ^2^ 0.997, B_M__ax_ 100 and Kd 66 ± 10 nmol/L. (D) Representative all‐points frequency histogram showing profound reduction in current at all levels corresponding to channel open states. Each bar represents a 0.1 pA bin width. Data from analysis of 20 sec segments of cell‐attached recordings.

The effects of PK‐THPP on TASK channel activity in type‐1 cells was studied over a range of concentrations from 40 to 400 nmol/L. Figure 3A and B show representative traces of channel activity in a cell attached patch from a type‐1 cell. Application of 400 nmol/L PK‐THPP caused a rapid and profound inhibition of native TASK channel activity by 89 ± 3% (*n* = 10; *P* = 0.034). Figure 3C shows the concentration dependence of PK‐THPP mediated type‐1 cell TASK channel inhibition which indicated a K_i_ of ~66 nmol/L. All‐points histograms revealed that PK‐THPP caused a reduction in channel activity across all conductance levels (Fig. 3D).

#### A1899

**Figure 4:**
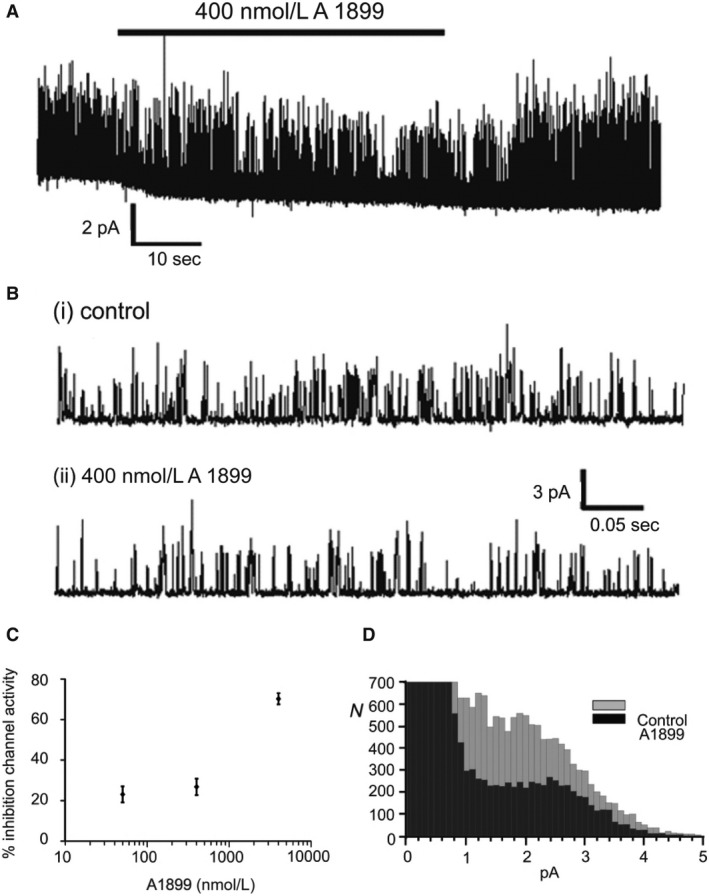
(A) Representative recording of type‐1 cell TASK channel activity over ~1.5 min of a cell‐attached patch recording demonstrating inhibition of channel activity by 400 nmol/L A1899. (B) Data from same recording over ~0.5 sec intervals under control conditions (i) and in the presence of A1899 (ii). (C) Concentration‐response relationship of effects of A1899 on type‐1 cell TASK channel activity (expressed as % inhibition in the presence of A1899 compared to control). Data are mean ± SEM (*n* = 4, 8 and 6 for 40, 400 and 4000 nmol/L concentrations of A1899, respectively). (D) Representative all‐points frequency histogram showing reduction of current at all levels corresponding to channel open states. Each bar represents a 0.1 pA bin width. Data were generated from analysis of 20 sec segments from cell‐attached recordings.

We studied the effects of A1899 over a range of concentrations from 50 to 4000 nmol/L. In cell attached patches in type‐1 cells 400 nmol/L A1899 caused a rapid and reversible inhibition of TASK channel activity by 34 ± 7% (*n* = 8; *P* = 0.028; Fig. 4A and B). A similar level of inhibition was also seen at a lower concentration of 50 nmol/L and a substantive further increase in inhibition at 4000 nmol/L to over 60% (see Fig. 4C). We did not observe saturation of the response to A1899 with the range of concentrations tested so have not attempted to define a K_i_. Figure 4D presents a representative all‐points histogram which shows that at 400 nmol/L A1899 causes an inhibition of TASK channel activity across all conductance levels.

### Effects of TASK channel inhibitors on Type‐1 cell [Ca^2+^]_i_ signalling

Having established that inhibitors of heterologously expressed homomeric TASK1 and TASK3 were also effective inhibitors of the endogenous TASK channels in type‐1 cells, which are predominantly TASK1‐TASK3 heterodimers, we next sought to determine whether they were effective stimulants of the carotid body as would be predicted. To study type‐1 cell excitation we employed measurements of calcium signaling which have been widely applied in the past to study both oxygen and acid sensing.

#### PK‐THPP

**Figure 5:**
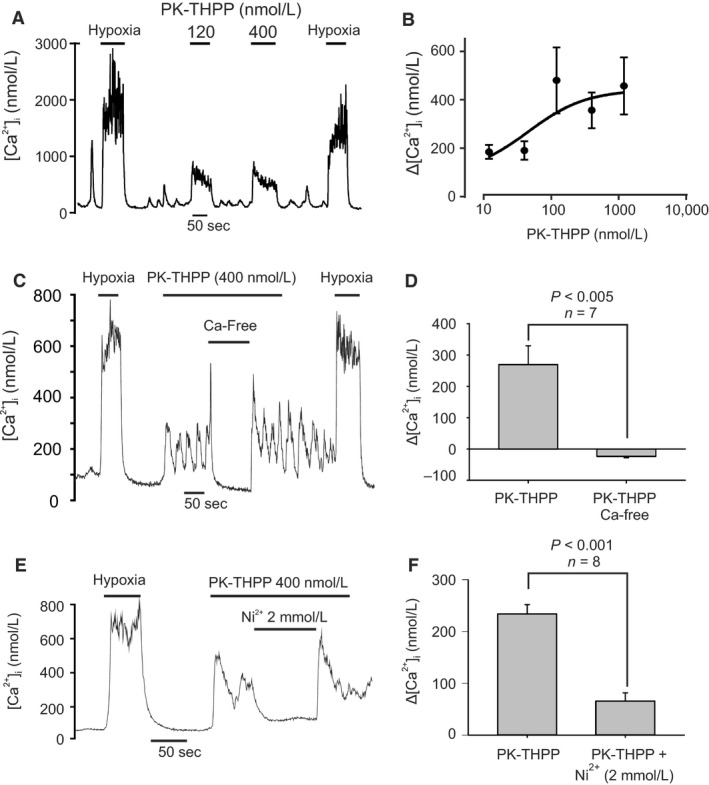
Effects of PK‐THPP on type‐1 cell [Ca^2+^]_i_. (A) Recording showing effects of application of two different concentrations (120 and 400 nmol/L) of PK‐THPP causing an abrupt rise in [Ca^2+^]_i_. (B) Concentration‐response graph of effects of PK‐THPP on [Ca^2+^]_i_. Each point represents mean ± SEM (*n* = 5–7) and the curve is fit by non‐linear least squares regression to the equation Δ[Ca^2+^]_i_ = a  +  b. [PK‐THPP]/(EC50 +  [PK‐THPP]). The estimated EC50 is 45.2 nmol/L and the *r* ^2^ for correlation is 0.19. (C) Effect of Ca^2+^ free Tyrode on [Ca^2+^]_i_ responses evoked by 400 nmol/L PK‐THPP. Note rapid reduction in [Ca^2+^]_i_ in the absence of external Ca^2+^. (D) Summary data showing effects of PK‐THPP on Δ[Ca^2+^]_i_ ([Ca^2+^]_i_ in the presence of PK‐THPP minus baseline [Ca^2+^]_i_) under normal conditions and in the absence of extracellular Ca^2+^. Data are mean ± SEM. Statistical comparison is a paired *t* test. (E) Effect of 2 mmol/L Ni^2+^, a voltage‐gated Ca^2+^‐channel inhibitor, on [Ca^2+^]_i_ responses evoked by 400 nmol/L PK‐THPP. Note rapid reduction in [Ca^2+^]_i_ upon application of Ni^2+^. (F) Summary data showing effects of PK‐THPP on Δ[Ca^2+^]_i_ under normal conditions and in the presence of Ni^2+^. Data are mean ± SEM. Statistical comparison is a paired *t* test.

Figure 5A shows that application of PK‐THPP evoked a rapid increase in glomus cell [Ca^2+^]_i_ in a manner similar to that of hypoxia. Hypoxia alone causes a rapid rise in [Ca^2+^]_i_ to a peak value followed by a modest decline. Some spontaneous spikes in the [Ca^2+^]_i_ trace were also evident in some type‐1 cells. PK‐THPP also elicited a rapid initial increase in [Ca^2+^]_i_ often followed by repetitive [Ca^2+^]_i_ “spiking,” the spikes almost returning to baseline in some cells. [Ca^2+^]_i_ responses to PK‐THPP were often slow to reverse following PK‐THPP removal taking minutes for [Ca^2+^]_i_ to return to baseline (see e.g., Fig. 5C). In assessing the concentration dependence of the effects of PK‐THPP on [Ca^2+^]_i_ we used the magnitude of the largest (which was always the first) spike in calculating the “response” to each level of PK‐THPP applied across a range of concentrations from 12 to 1200 nmol/L. The concentration – [Ca^2+^]_i_ response relationship was nonlinear (Fig. 5B) and appeared to show saturation at higher levels of PK‐THPP with an estimated EC50 of 45 nmol/L. There was a significant correlation between the concentration of PK‐THPP and [Ca^2+^]_i_ (Spearman's Rho = 0.359, *P* = 0.034).

PK‐THPP evoked changes in [Ca^2+^]_i_ were abolished in Ca^2+^‐free solution containing 100 *μ*mol/L EGTA (Fig. 1C and D), confirming that the effects of PK‐THPP were due to influx of external Ca^2+^. The PK‐THPP induced rise in [Ca^2+^]_i_ was also reversed by the voltage‐gated Ca^2+^‐channel inhibitor Ni^2+^ (2 mmol/L, see Fig. 5E and F) indicating that membrane depolarization and voltage‐gated Ca^2+^‐entry was the most likely cause of the PK‐THPP induced rise in [Ca^2+^]_i._

#### A1899

**Figure 6:**
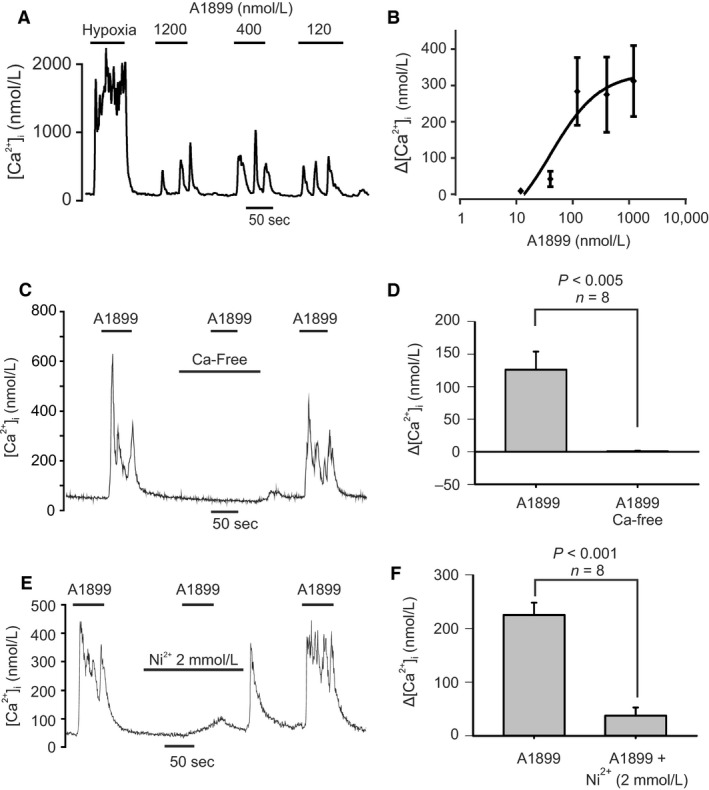
Effects of A1899 on type‐1 cell [Ca^2+^]_i_. (A) Effects of application of three different concentrations (120, 400 and 1200 nmol/L) of A1899 demonstrating an abrupt, spiking, rise in [Ca^2+^]_i_. (B) Concentration‐response graph of effects of A1899 on [Ca^2+^]_i_. Each point represents mean ± SEM. The curve is fit by non‐linear least squares regression to the equation Δ[Ca^2+^]_i_ = a + b. [A1899]/(EC50 +  [A1899]). The estimated EC50 is 40.8 nmol/L and the *r* ^2^ for correlation is 0.23. (C) Effect of Ca^2+^ free Tyrode on [Ca^2+^]_i_ responses evoked by 400 nmol/L A1899. Note absence of any [Ca^2+^]_i_ response to A1899 when applied in Ca^2+^ free Tyrode. (D) Summary data showing effects of A1899 on Δ[Ca^2+^]_i_ ([Ca^2+^]_i_ in the presence of A1899 minus baseline [Ca^2+^]_i_) under normal conditions and in the absence of extracellular Ca^2+^.Statistical comparison is a paired *t* test. (E) Effect of 2 mmol/L Ni^2+^, a voltage‐gated Ca^2+^‐channel inhibitor, on [Ca^2+^]_i_ responses evoked by 400 nmol/L A1899. Note much smaller and slower rise in [Ca^2+^]_i_ when A1899 is applied in the presence of Ni^2+^. (F) Summary data showing effects of A1899 on Δ[Ca^2+^]_i_ under normal conditions and in the presence of Ni^2+^. Data are mean ± SEM. Statistical comparison is a paired *t* test.

Figure 6A shows the effects of A1899 to be very similar to that of PK‐THPP, that is, application of A1899 led to a rapid rise in [Ca^2+^]_i_ which often returned towards base line followed by a succession of similar [Ca^2+^]_i_ spikes which continued until the drug was withdrawn. Unlike PK‐THPP [Ca^2+^]_i_ responses to A1899 were almost always rapidly reversed/ablated upon drug withdrawal. There was a positive correlation between A1899 concentration and [Ca^2+^]_i_ response (Spearman's Rho = 0.651, *P* < 0.001) which was also nonlinear such that at the lower doses (<24 nmol/L) there was minimal rise in [Ca^2+^]_i_ evoked by A1899 but a concentration‐dependent increase in [Ca^2+^]_i_ at higher concentrations with the response appearing to saturate at concentrations of 400 nmol/L and above).

As with PK‐THPP, the increase in [Ca^2+^]_i_ evoked by A1899 was abolished when cells were superfused in a Ca^2+^‐free EGTA solution (Fig. 6C and D) and inhibited substantially in the presence of 2 mmol/L Ni^2+^ (Fig. 6E and F). These observations again indicating that membrane depolarization and voltage‐gated Ca^2+^‐entry was the most likely cause of the A1899 induced rise in [Ca^2+^]_i._

#### ML365

**Figure 7:**
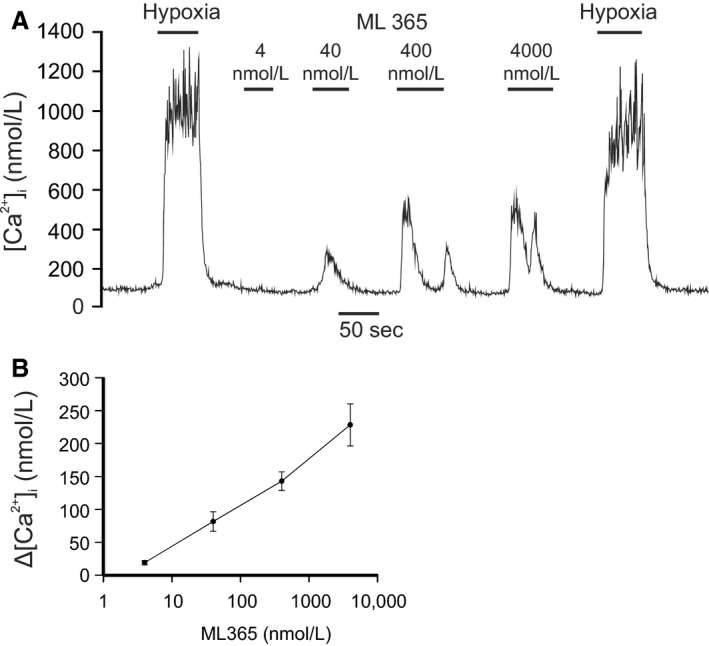
Effects of ML365 on type‐1 cell [Ca^2+^]_i_. (A) Representative trace showing effects of application of four different concentrations (4, 40, 400, and 4000 nmol/L) of ML365 demonstrating an abrupt rise in [Ca^2+^]_I_ in response to this TASK‐1 channel inhibitor. (B) Concentration‐response graph of effects of A1899 on [Ca^2+^]_i_. Each point represents mean ± SEM (*n* = 8).

Recently another compound, ML365, has been described as an inhibitor of TASK‐1 and TASK‐3 with 60‐fold selectivity for TASK‐1 over TASK‐3 (EC_50_'s 16 nmol/L and 1 *μ*mol/L, respectively, (Flaherty et al. 2014)). Although we have not yet tested this compound on channel activity in the type‐1 cell it seems reasonable to assume that as the identity of these channels is now confirmed other inhibitors of TASK‐1 and TASK‐3 should also function as stimulants of the type‐1 cell. Consequently, we tested the effects of ML365 over a range of concentrations (from 4 nmol/L to 4000 nmol/L). For these experiments the [Ca^2+^]_i_ response was averaged over approx. 40–50 sec of exposure to the drug. ML365 caused a significant and dose‐dependent increase in [Ca^2+^]_i_ (*P* < 0.001 Friedman repeated measures ANOVA on ranks) see Figure 7. The [Ca^2+^]_i_ response to ML365 did not appear to saturate over the range of concentrations tested. Varying degrees of spiking in the [Ca^2+^]_i_ trace were observed at different concentrations of ML365. Responses to ML365, where present, were rapid in onset and rapidly reversible upon drug removal.

### Doxapram, an established respiratory stimulant, also inhibits type‐1 cell TASK channels and evokes increase in cytosolic calcium

**Figure 8:**
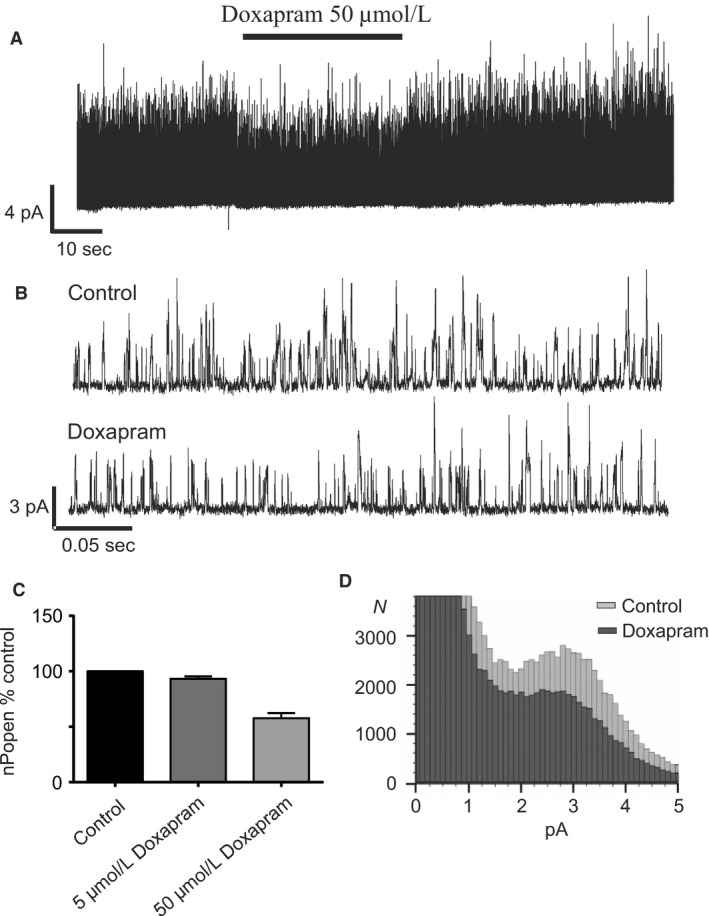
(A) Representative trace of Type‐1 cell TASK channel activity in a cell attached patch over ~1.5 min of recording, demonstrating reduction in activity by Doxapram. (B) Data from same recording over ~0.4 sec intervals under control conditions (i) and in the presence of doxapram (ii). (C) Concentration‐response relationship of doxapram on Type‐1 cell TASK channel activity. nPopen is plotted as % of that observed under control conditions. Doxapram was applied at 5 *μ*mol/L (*n* = 3) and 50 *μ*mol/L (*n* = 7). (D) Representative all‐points frequency histogram showing doxapram decreased all current levels corresponding to open channel activity (**e**ach bar represents a 0.1 pA bin width, data generated from 20 sec segments of cell‐attached recordings).

**Figure 9:**
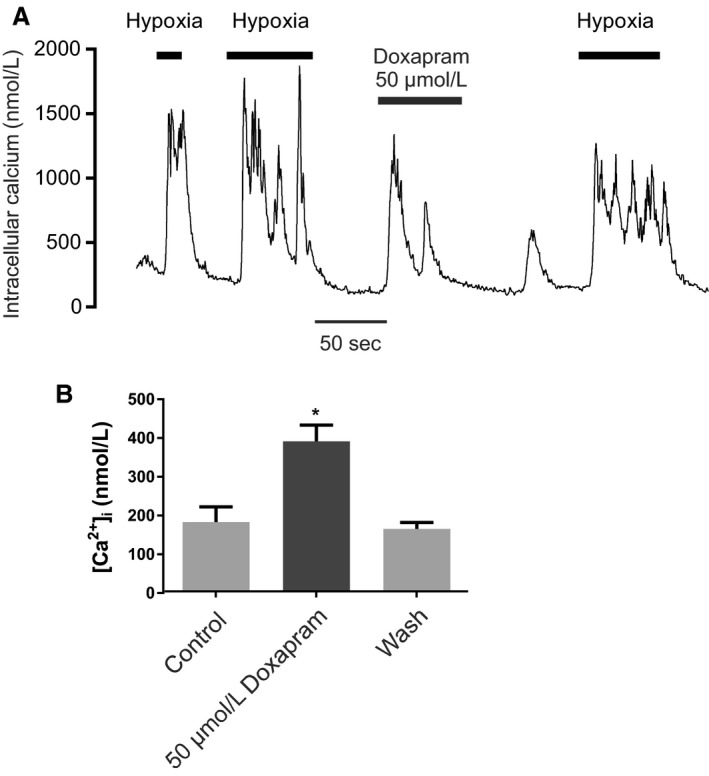
(A) Representative trace of the effect doxapram on glomus cell [Ca^2+^]i. (B) Summary of quantitative effect of doxapram on glomus cell [Ca^2+^]i. data are mean ± SEM *n* = 8, *P* < 0.001 (doxapram vs. control, paired *t* test).

Doxapram is a widely used respiratory stimulant that is also reported to inhibit cloned TASK channels (Cotten et al. 2006). It is consequently thought likely that doxapram exerts some of its effects by inhibiting native TASK channels in the type‐1 cell and thus exciting the type‐1 cell. In view of the results obtained above with other TASK channel inhibitors we next sought to investigate the effects of doxapram on type‐1 cells. Figure 8 demonstrates that at high (50 *μ*mol/L) levels doxapram does indeed inhibit type‐1 cell TASK channel activity rapidly and reversibly by 42.3 ± 4.6% (*n* = 7, *P* = 0.018, Fig. 8C). This effect was observed across all conductance levels (Fig. 8D). 50 *μ*mol/L doxapram also caused a rapid and reversible increase in [Ca^2+^]_i_ (Fig. 9).

### Interaction of TASK channel inhibitors with BK_Ca_ and delayed rectifier K‐channel inhibitors

**Figure 10:**
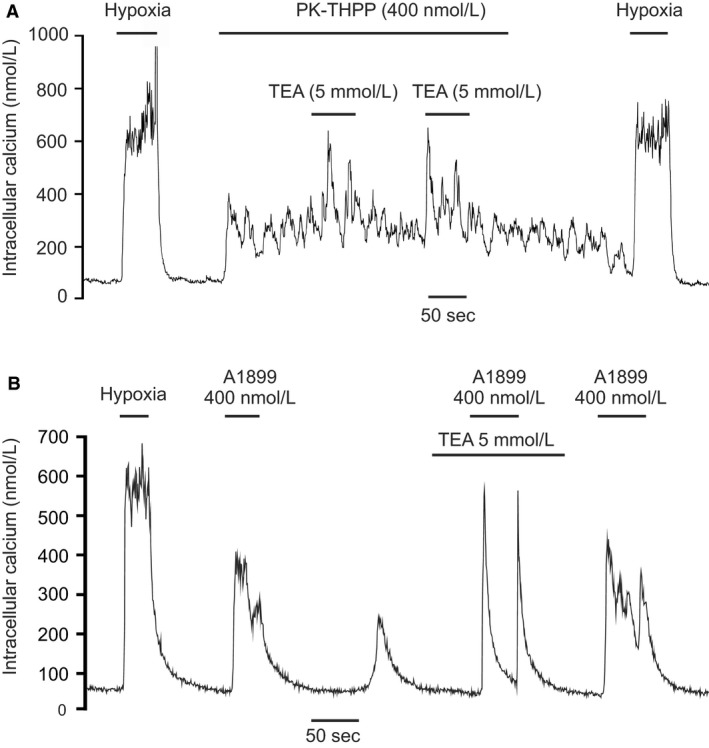
Effects of 5 mmol/L TEA on the [Ca^2+^]_i_ response to TASK channel inhibitors. (A) TEA + 400 nmol/L PK‐THPP. (B) TEA + 400 nmol/L A1899. Note increase in size of [Ca^2+^]_i_ spikes in the presence of TEA.

**Figure 11:**
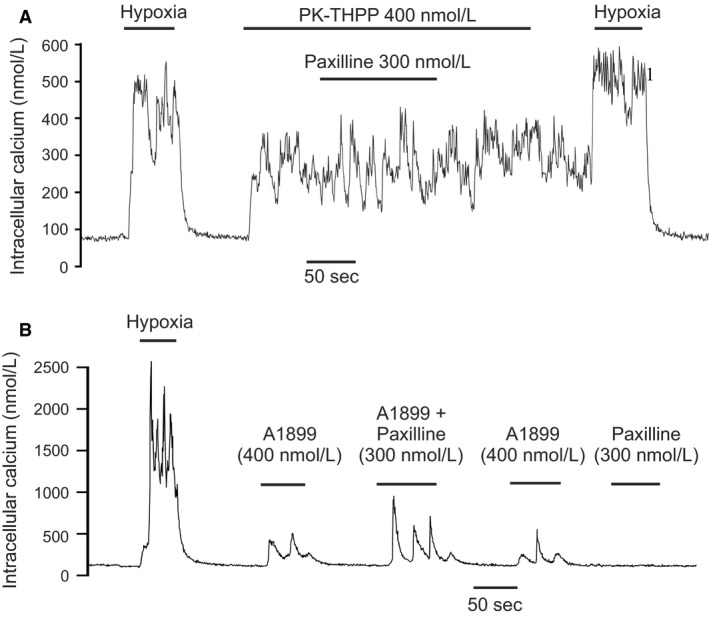
Effects of 300 nmol/L paxilline on the [Ca^2+^]_i_ response to TASK channel inhibitors. (A) Paxilline + 400 nmol/L PK‐THPP. (B) Paxilline + 400 nmol/L A1899. Note increase in [Ca^2+^]_i_ response to A1899 during application of paxilline but that paxilline has no appreciable effects on the response to PK‐THPP.

**Figure 12:**
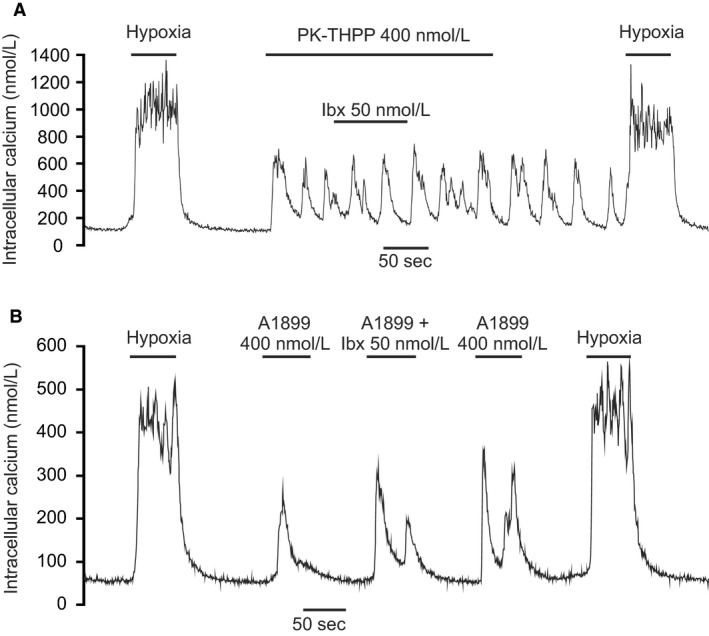
Effects of 50 nmol/L Iberiotoxin on the [Ca^2+^]_i_ response to TASK channel inhibitors. (A) Iberiotoxin + 400 nmol/L PK‐THPP. (B) Iberiotoxin + 400 nmol/L A1899. Note apparent lack of effect of iberiotoxin in presence of PK‐THPP.

Although TASK channels appear to contribute to the majority of background K‐channel activity around the resting potential they may not be the only channels directly involved in mediating the cellular response to hypoxia. A number of other potassium channels have been reported to also be oxygen sensitive in type‐1 cells, and although not particularly active at resting membrane potentials it is thought that they become active as the cell depolarizes and/or as intracellular calcium rises (Wang and Kim 2017). Thus, the hypoxic modulation of these channels may contribute to the overall [Ca^2+^]_i_ ‐response to hypoxia even though they cannot initiate that response (see discussion). In the rat type‐1 cell the only other oxygen‐sensitive K‐channel thus far reported is the large conductance calcium activated K channel (BK_Ca_) (Peers 1990a). We noted in our study of TASK channel inhibitors that whilst all were capable raising [Ca^2+^]_i_ in type‐1 cells rarely did that effect match or exceed the [Ca^2+^]_i_ response to hypoxia. This suggests that hypoxic modulation of other channels might also be needed in order to generate a full response (see discussion). We therefore thought to test the hypothesis that inhibition of BK_Ca_ and/or delayed rectifier K^+^ channels could augment [Ca^2+^]_i_ response to TASK inhibition. In this study, we used both A1899, a relatively mild inhibitor of type‐1 cell TASK channel activity (at 400 nmol/L, see Fig. 4C), as well as PK‐THPP a stronger inhibitor of type‐1 cell TASK (also at 400 nmol/L see Fig. 3C). As A1899 is rapidly reversible, BK_Ca_ channel inhibitors were added coincidently with the application of A1899. For PK‐THPP we allowed the response to PK‐THPP to stabilize first before adding the BK_Ca_ inhibitor in the continued presence of PK‐THPP (see Figs. 10, 11, 12). The drugs tested were TEA (5 mmol/L) a nonselective inhibitor of delayed rectifier K‐channels and BK_Ca_ in type‐1 cells, paxilline a selective BK_Ca_ inhibitor (Knaus et al. 1994; Sanchez and McManus 1996) and iberiotoxin also a selective inhibitor of BK_Ca_ (Candia et al. 1992). To quantify the effects of these drugs we measured average [Ca^2+^]_i_ responses which were defined as the increase in [Ca^2+^]_i_ during periods in which the cell was exposed to K‐channel inhibitors relative to base line [Ca^2+^]_i_ prior to the application of potassium channel inhibitors. For studies using PK‐THPP this was applied first for at least 50 sec followed by co‐application of a BK_Ca_‐channel inhibitor for >= 50 sec and then removal of BK_Ca_ channel inhibitor (see e.g., Fig. 10A). In this protocol, the control response to PK‐THPP alone was determined over a 50 sec period during application of PK‐THPP but before application of BK_Ca_ inhibitor. The response to PK‐THPP plus BK_Ca_ inhibitor was then determined over a 50 sec time period in which both drugs were present. For studies using A1899 this drug was applied on its own first and then in combination with a BK_Ca_ channel inhibitor (test). Each application of drug lasted >= 50 sec and was followed by a return to baseline conditions (see e.g., Fig. 10B).

#### TEA

**Figure 13:**
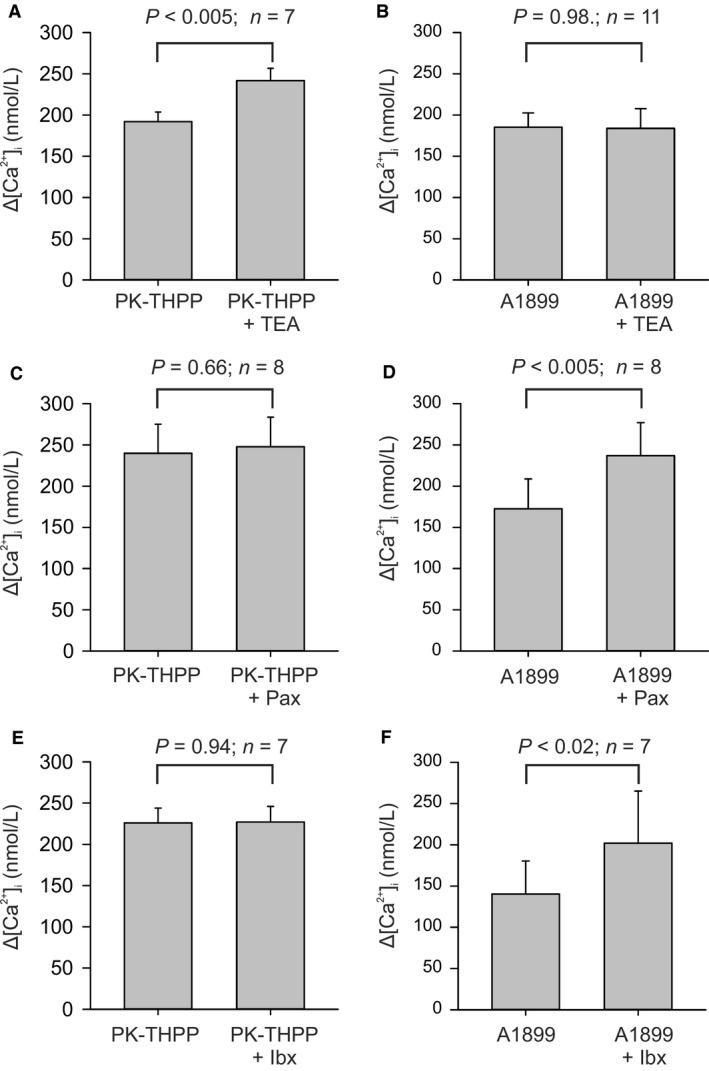
Summary of effects of combinations of K‐channel inhibitors on intracellular calcium. Y axis values represent the increase in [Ca^2+^]_i_ (over base line [Ca^2+^]_i_) in the presence of K^+^‐channel inhibitor/s. [Ca^2+^]_i_ was averaged over the duration of exposure to the inhibitors (approx. 40–50 s). Experimental protocols are shown in Figures 8,9 and 10. Inhibitor concentrations were PK‐THPP 400 nmol/L, A1899 400 nmol/L, TEA 5 mmol/L, Paxilline (Pax) 300 nmol/L, and Iberiotoxin (Ibx) 50 nmol/L. Values are mean ± SEM,* P* values were calculated using a paired *t* test in all panels except (F) which uses a Wilcoxon signed‐rank test.

At 5 mmol/L TEA increased the amplitude of [Ca^2+^]_i_ spiking activity in the presence of 400 nmol/L PK‐THPP and increased the average [Ca^2+^]_i_ measured over an approximate 50‐sec time period before and during TEA application (see Figs. 10A and 13A). This response to TEA was modest in amplitude and rapidly reversible. Addition of TEA (5 mmol/L) and A1899 (400 nmol/L) together did not significantly increase the average [Ca^2+^]_i_ response compared to the actions of A1899 alone. It was however noted that the peak level of [Ca^2+^]_i_ observed in the presence of TEA + A1899 was invariably greater than that observed in the presence of A1899 alone (666 ± 128 nmol/L vs. 871 ± 166; *n* = 11, *P* < 0.02) see Figures 10B and 13B.

#### Paxilline

At 300 nmol/L paxilline had no significant effect on the [Ca^2+^]_i_ response to 400 nmol/L PK‐THPP (Figs. 11A and 13C) but did increase the average [Ca^2+^]_i_‐response to 400 nmol/L A1899 (Figs. 11B and 13D).

#### Iberiotoxin

At 50 nmol/L iberiotoxin had no discernible, or statistically significant, effect on the average [Ca^2+^]_i_ response to PK‐THPP (400 nmol/L) but did cause a small increase in the response to A1899 (400 nmol/L) see Figures 12, 13E and F.

### Comparison of the effects of K‐channel inhibitors with those of hypoxia

**Figure 14:**
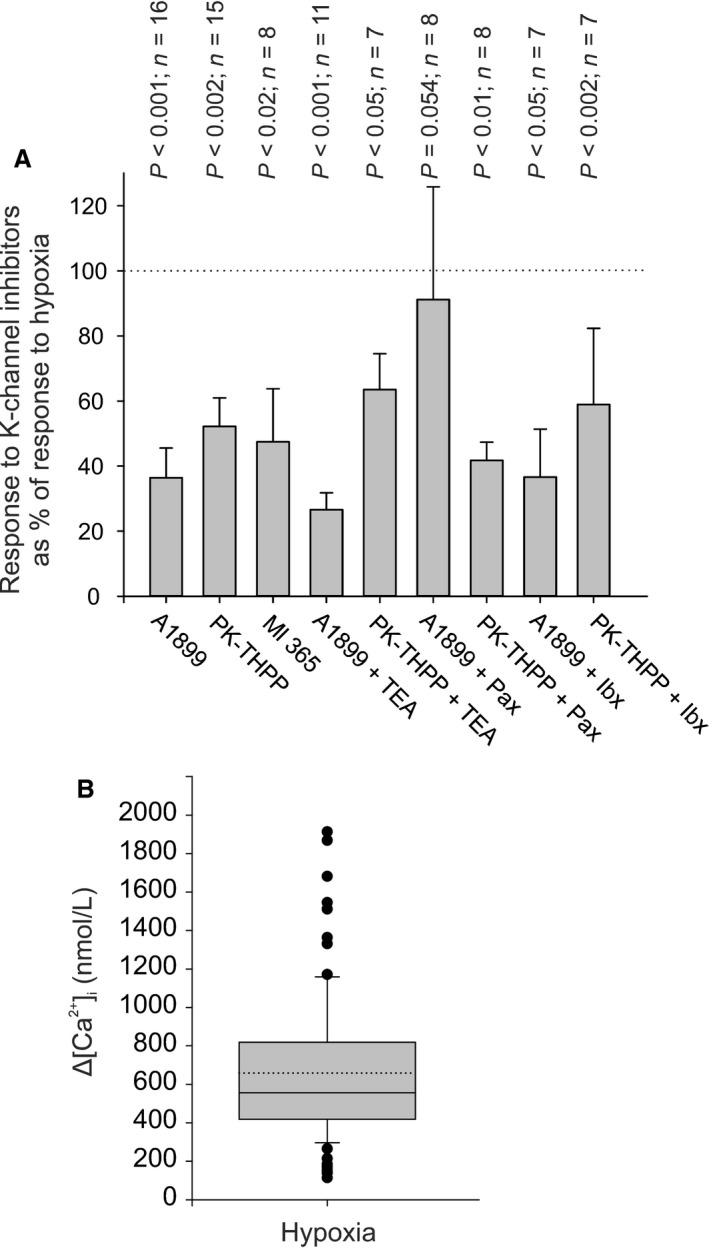
(A) Comparison of effects of K‐channel inhibitors with those of hypoxia (N_2_ –equilibrated Tyrode see methods). Data (y axis) represents the average [Ca^2+^]_i_ response recorded in the presence of K^+^ channel inhibitor/s expressed as a percentage of the response to a hypoxic stimulus recorded in the same cell/s. The [Ca^2+^]_i_ response to inhibitor/hypoxia was determined from the average [Ca^2+^]_i_ recorded over an approx. 50 sec interval in the presence of inhibitor/hypoxia minus baseline [Ca^2+^]_i_ (recorded over 50 sec period under control conditions). Data are mean ± SEM. *P* values represent a comparison of response to inhibitor versus hypoxia using a paired t‐test. Note in all conditions bar one (which only narrowly failed to reach significance) the reponse to hypoxia was always greater than that to any single TASK‐channel inhibitor or combination of TASK and BK_C__a_ channel inhibitor. Drug concentrations were PK‐THPP 400 nmol/L, A1899 400 nmol/L, Ml365 4 *μ*mol/L, TEA 5 mmol/L, paxilline (Pax) 300 nmol/L, and iberiotoxin (Ibx) 50 nmol/L. (B) Box and whisker plot of all responses to hypoxia recorded in this study (*n* = 87). Box 25th‐75th percentiles, whisker 10th and 90th percentiles, broken line = mean, solid line = median. Individual outlying values only are plotted.

In each of the experiments conducted using K‐channel inhibitors type‐1 cells were first exposed to a strong hypoxic stimulus to confirm their identity and oxygen sensitivity. This has allowed us to make direct comparisons between the [Ca^2+^]_i_ response to hypoxia and the [Ca^2+^]_i_ response to potassium channel inhibitor (or combination of inhibitors). Figure 14A presents the inhibitor response as a percentage of the hypoxic response. The size of the response to K^+^‐channel inhibitor/s was also compared to the response to hypoxia by paired t‐test (data presented in Fig. 14A). It is clear that for all inhibitors/combinations of inhibitor bar one the [Ca^2+^]_i_ response to inhibitors is significantly less than that to hypoxia. In the one exception, A1899 +  paxilline, which only narrowly failed to reach significance, the data set contained three cells with very low responses to hypoxia which fell within the group of outliers depicted in Figure 14B. This suggests that none of the inhibitor combinations were truly able to match the effects of hypoxia.

**Figure 15:**
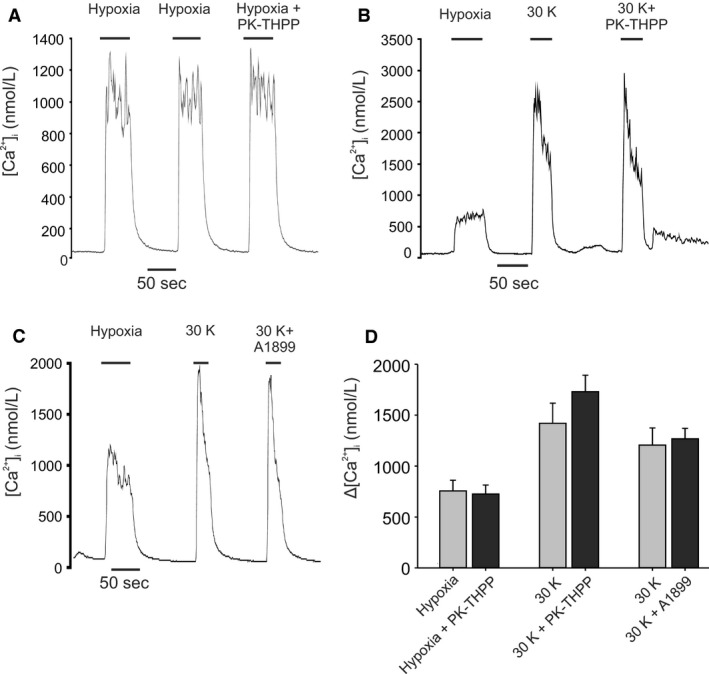
Effects of TASK channel inhibitors on calcium responses to high potassium and to hypoxia. (A) Representative trace of cellular calcium response to hypoxia alone, and in the presence of 400 nmol/L PK‐THPP. (B) Calcium response to depolarization by 30 mmol/L K^+^ alone, and in the presence of 400 nmol/L PK‐THPP. Increase in K^+^ was osmotically compensated for by reduction in Na^+^. (C) Calcium response to depolarization by 30 mmol/L K^+^ alone, and in the presence of 400 nmol/L A1899. (D) Summary of effects of TASK channel inhibitors on the calcium response to depolarization with 30 mmol/L K^+^ and the effects of PK‐THPP on the [Ca^2+^]_i_ response to hypoxia. Comparisons are i) Hypoxia versus Hypoxia + 400 nmol/L PK‐THPP,* n* = 6, *P* = 0.36, ii) 30 mmol/L K^+^ versus 30 mmol/L K^+^ + 400 nmol/L PK‐THPP,* n* = 7, *P* = 0.07, iii) 30 mmol/L K^+^ versus 30 mmol/L K^+^ + 400 nmol/L A1899, *n* = 6, *P* = 0.63.

In view of the above observation, we also tested the effects of A1899 and PK‐THPP on voltage‐gated Ca^2+^ entry in type‐1 cells in response to depolarization with high (30 mmol/L) K^+^ in order to exclude the possibility that these drugs might have previously unknown effects on voltage gated Ca‐channels. Neither A1899 nor PK‐THPP reduced the [Ca^2+^]_i_ response to 30 mmol/L K^+^ (Fig. 15). These drugs do not therefore appear to interfere with voltage gated Ca^2+^ entry/signaling per se. We further tested the effects of PK‐THPP on the response to hypoxia to see if it might interfere with any other aspect of signaling involved in mediating the [Ca^2+^]_i_‐response to hypoxia/TASK inhibition. We found that 400 nmol/L PK‐THPP had no discernible effect on [Ca^2+^]_i_ signaling in response to hypoxia (figure 15A). The failure of PK‐THPP and A1899 to fully match the effects of hypoxia cannot therefore be ascribed to some unknown, nonspecific, inhibitory effect upon Ca‐signaling.

## Discussion

We and others have previously described abundant potassium channel activity in the membranes of carotid body type‐1 cells at resting membrane potentials (Buckler et al. 2000; Kim et al. 2009). These channels are characterized by very flickery kinetics with short and variable amplitude open states (Williams and Buckler 2004). They are thought to mediate the background, or leak, potassium conductance/current in type‐1 cells which plays a major role in setting the cells resting membrane potential and in bringing about membrane depolarization in response to a number of chemostimuli including hypoxia, acidosis, and metabolic poisons (Buckler 2015). These channels are widely believed to belong to the TASK channel subgroup of the tandem‐p‐domain potassium channel family. Specifically they are thought to be a mixed population comprising primarily of TASK‐1‐TASK‐3 heterodimers together with a lesser number of TASK‐1 and TASK‐3 homodimers. This attribution has been based on the biophysical properties of both whole cell currents and single channel recordings as well as studies using mice in which *Task‐1* and/or *Task‐3* genes have been disrupted (Kim et al. 2009; Turner and Buckler 2013). Compelling pharmacological confirmation of the identity of these channels has, however, been lacking due to the poor specificity of agents that were originally used to characterize these types of potassium channel. The lack of selective inhibitors for these endogenous channels has also hindered research into their functional relevance. Here, we demonstrate that selective inhibitors of TASK‐1 and TASK‐3 also inhibit endogenous TASK channels in the type‐1 cell and are effective stimulants of type‐1 cell [Ca^2+^]_i_ signaling.

A1899 is reported to be a selective inhibitor of TASK‐1 showing modest, approx. 10 fold, selectivity over TASK‐3 (IC_50_ 35 nmol/L vs. 318 nmol/L) and much greater selectivity over other tandem‐p‐domain K‐channels (Streit et al. 2011; Kiper et al. 2015). A1899 is an open pore blocker and in consequence its apparent IC_50_ may depend on potassium ion concentration. In our studies, we used high potassium in the pipette filling solution whilst recording in the cell attached patch configuration and achieved 63% inhibition of heterologously expressed TASK‐1 channel activity in HEK 293 cells with 400 nmol/L A1899. This compares favorably with the level of inhibition for 400 nmol/L A1899 reported in high extracellular K^+^ in oocytes (59.7%; (Streit et al. 2011)). PK‐THPP is a potent inhibitor of TASK‐3 channels (compound 23 in (Coburn et al. 2012)) with modest (10‐fold) selectivity for TASK‐3 over TASK‐1 (reported IC_50_ values of 35 nmol/L for TASK‐3 and 300 nmol/L for TASK‐1). It does however show good selectivity over TREK and some other tandem‐p‐domain K‐channels (Coburn et al. 2012). Effects of high K on the inhibitory potency of PK‐THPP have not been reported. In our hands, however, we obtained strong (85%) inhibition of heterologously expressed TASK‐3 in HEK cells with 400 nmol/L PK‐THPP. Both inhibitors are therefore effective under the recording conditions used in this study (cell attached patch configuration).

In studies conducted on type‐1 cells we found that both A1899 and PK‐THPP inhibited TASK channel activity in a dose‐dependent manner. With A1899 there was only a modest inhibition up to 400 nmol/L but with a greater degree of inhibition at much higher concentrations. This may suggest multiple sites of action. In principle we would expect three potential targets homomeric TASK‐1, homomeric TASK‐3 and heteromeric TASK‐1/TASK‐3. The effect of A1899 on TASK‐1/TASK‐3 heterodimers has thus far only been studied in Xenopus Oocytes. Here, A1899 was shown to be much less effective at blocking TASK‐1/TASK‐3 heterodimers compared to TASK ‐1 homodimers (Rinné et al. 2015). The relatively weak effects of A1899 at concentrations up to 400 nmol/L is therefore consistent with the expected preponderance of TASK‐1/TASK‐3 heterodimers in the type‐1 cell (Kim et al. 2009; Turner and Buckler 2013).

In contrast PK‐THPP produced a robust, dose‐dependent, saturating, inhibition of TASK channel activity of up to 90% at 400 nmol/L. This matches the level of inhibition seen in heterologously expressed TASK‐3 channels and, given that the majority of TASK channels in the type‐1 cell membrane are thought to be heterodimers, indicates that this compound is at least as effective at inhibiting TASK‐1/TASK‐3 heterodimers as it is at inhibiting TASK‐3. Our estimated IC_50_ for inhibition of the type‐1 cell channels was approx. 66 nmol/L.

The observation that both A1899 and PK‐THPP block background channels in the type‐1 cell provides further confirmation that these channels are indeed TASK channels. Moreover, these agents provide new opportunities to explore the functional significance of TASK channels in controlling type‐1 cell excitability. A key element of the signaling mechanisms responsible for mediating a depolarizing response to natural chemostimuli (acidosis and hypoxia) is the inhibition of potassium channels. Selective inhibitors of potassium channels give us an opportunity to unpick the specific roles of individual channel types. Our current model is that calcium entry is promoted by depolarization through the inhibition of the background K‐current (of which TASK is presumed to be the main contributor) but may then be sustained and or enhanced via the concurrent inhibition of calcium activated potassium currents. The obvious prediction that derives from this model is that inhibition of TASK/background K‐current should be sufficient to at least evoke an initial rise in intracellular calcium via voltage‐gated Ca‐entry.

We found that A1899 and PK‐THPP both increased intracellular calcium in isolated type‐1 cells in a concentration‐dependent manner. This rise in calcium was prevented, or reversed in calcium‐free medium and was inhibited by the voltage‐gated Ca^2+^‐channel inhibitor Nickel (2 mmol/L). These data support the hypothesis that inhibition of TASK channel activity in type‐1 cells is capable of initiating voltage gated calcium entry. We further confirmed this with another more recent TASK inhibitor ML365. ML365 is a selective TASK‐1 inhibitor, but also inhibits TASK3 (IC50's = 16 nmol/L TASK‐1 and 990 nmol/L TASK‐3) (Flaherty et al. 2014). ML365 also evoked a dose dependent increase in intracellular calcium in the type‐1 cell. We noted however that although A1899, PK‐THPP, and ML365 could evoke a robust increase in [Ca^2+^]_i_ this was, in almost all cases, lower that that seen in response to hypoxia in the same cells/recordings (see Fig. 14). Thus, although 400 nmol/L PK‐THPP caused 90% inhibition of type‐1 cell TASK channel activity it did not produce as robust a calcium response as did hypoxia which inhibits TASK channel activity by around 40–80% (Buckler et al. 2000; Kim et al. 2009).

As discussed above TASK channels are not the only potassium channels reported to be oxygen sensitive in type‐1 cells. In the rat type‐1 cell a large conductance calcium activated potassium channel has been repeatedly reported to be oxygen sensitive (Peers 1990a; Wyatt and Peers 1995; Riesco Fagundo et al. 2001), although this has recently been disputed (Wang and Kim 2017). This channel is no longer thought to be particularly active at the resting membrane potential in normoxia but may become active as the cell is depolarized (Wang and Kim 2017). Consequently, BK_Ca_ channel inhibitors alone seem to have little effect on basal [Ca^2+^]_i_ in freshly isolated rat type‐1 cells (Buckler 1997) unless they are partially depolarized (Wang and Kim 2017). BK_Ca_ and Kv channels may, however, become active as the cell depolarizes and [Ca^2+^]_i_ rises thus acting as a brake on cell excitation. The inhibition of such channels by hypoxia (or acidosis) could therefore be argued to play a role in mediating the calcium response to chemostimuli by reducing the extent to which these channels oppose cell depolarization and elevation of [Ca^2+^]_i_. This idea is supported by the observation that some BK_Ca_ and K_V_ channel inhibitors can augment the [Ca^2+^]_i_ response to hypoxia (Buckler 1997; Wang and Kim 2017). We therefore questioned whether the smaller [Ca^2+^]_i_ response to direct TASK channel inhibition (when compared to hypoxia) might be due to the lack of concurrent inhibition of BK_Ca_ channel activity. In order to test this hypothesis we studied the effects of inhibiting both BK_Ca_ channels and TASK channels at the same time. In our studies we found that 5 mmol/L TEA did indeed modestly augment the [Ca^2+^]_i_ response to PK‐THPP and also altered the response to A1899 (in so far as peak [Ca^2+^]_i_ was increased but not mean [Ca^2+^]_i_). This is consistent with the expectation that as inhibition of TASK channels leads to depolarization so other types of voltage, and or calcium activated K‐channels may become active limiting the extent of depolarization and thus curb the rise in [Ca^2+^]_i_. TEA is not, however, selective for BK_Ca_ as it also inhibits much of the delayed rectifier K‐current in type‐1 cells. Iberiotoxin (a selective BK_Ca_‐inhibitor with an estimated IC 50 of 250 pmol/L (Galvez et al. 1990)) did not appear to have any effect upon the [Ca^2+^]_i_ response to PK‐THPP but did seem to slightly augment the response to A1899. The same pattern was seen with paxilline another BK_Ca_‐channel inhibitor which slightly augmented the [Ca^2+^]_i_ ‐response to A1899 but not that to PK‐THPP. The relatively small effect of these BK_Ca_ channel inhibitors was surprising given the proposed role for BK_Ca_ but is not inconsistent with recent studies on the effects of K‐channel inhibitors on the response to hypoxia in which TEA and 4AP enhanced [Ca^2+^]_i_ but iberiotoxin did not(Wang and Kim 2017). It is difficult to speculate on why the effects of BK_Ca_ inhibitors are so variable. It may depend on the precise nature of the electrical signals being produced. Freshly isolated rat type‐1 cells have a resting membrane potential of around −60 mV (in perforated patch recordings) and a low resting [Ca^2+^]_i_. This situation is mostly stable although occasional spontaneous depolarizations and [Ca^2+^]_i_ spikes are seen. In response to hypoxia, hypercapnia, metabolic acidosis, and mitochondrial inhibitors type‐1 cells undergo a rapid depolarization producing a receptor potential which promotes action potential generation. These action potentials may be tonic or bursting. With powerful stimuli, for example, anoxia and mitochondrial inhibitors the receptor potential may be large and action potentials reduced to small (10–20 mV) spikes (Buckler and Vaughan‐Jones 1994a; Buckler and Vaughan‐Jones 1994b; Buckler 1997; Buckler and Vaughan‐Jones 1998; Buckler et al. 2000; Wyatt and Buckler 2004; Buckler 2012). The pattern of electrical activity may therefore vary with stimulus type and severity. This could affect the involvement, and influence, of BK_Ca_ channels. In addition, cell‐cell and animal‐animal variability may be present (see e.g.. Fig. 14 regarding variability of [Ca^2+^]_i_ response to hypoxia).

Irrespective of the reasons for the seemingly small influence of BK_Ca_ inhibitors, what is clear from these studies is that concomitant inhibition of the only two known oxygen‐sensitive channels in rat type‐1 cells fails to match the response to hypoxia itself. Thus, our current model of oxygen sensing is presumably incomplete. There may be other, as yet unidentified, oxygen‐sensitive currents/channels which contribute to membrane depolarization and the elevation of cytosolic calcium besides TASK and BK_Ca_. This is consistent with the observation that in *Task‐1:Task‐3* double knockout mice it is possible to identify type‐1 cells that retain the capability to produce robust [Ca^2+^]_i_ response to hypoxia (Turner and Buckler 2013). There is a limit to how far we can speculate as to the nature of such channels/currents except that they are probably not strongly inhibited by TEA and that they could be very small (a few pA) since the inhibition of TASK channels substantially reduces membrane conductance. An alternative possibility is that hypoxia enhances [Ca^2+^]_i_ signaling by interfering with mitochondrial Ca^2+^‐buffering. In many cells voltage‐gated Ca^2+^‐entry can result in large amounts of Ca^2+^ being taken up into the mitochondria thus buffering the rise in cytosolic [Ca^2+^]_i_ (Rizzuto et al. 2012). Mitochondrial Ca‐uptake depends upon the mitochondrial membrane potential. In type‐1 cells mitochondria depolarize in response to hypoxia (Buckler and Turner 2013). Thus, diminished mitochondrial Ca^2+^‐buffering could explain the greater rise in [Ca^2+^]_i_ seen in hypoxia compared to that seen with just K‐channel inhibition. Further investigation of oxygen sensitive currents and Ca^2+^‐signaling/buffering in the type‐1 cell would now seem warranted and feasible given the availability of selective and highly effective TASK channel inhibitors to help isolate other membrane currents.

We have not sought to compare the effects of acidosis on [Ca^2+^]_i_ with those of TASK‐channel inhibitors in this study. [Ca^2+^]_i_ – responses to moderate acidosis are generally weaker than the response to intense hypoxia (e.g., see (Dasso et al. 2000)) so there is no obvious reason to presuppose that excitation of type‐1 cells by acidosis needs the involvement of anything other than TASK channel inhibition. It is however of interest to note that BK_Ca_‐channels are also acid sensitive (Peers 1990b) and appear to be involved, alongside TASK, in acid sensing in mouse chromaffin cells. Indeed in chromaffin cells the combination of A1899 and paxilline most closely simulates the effects of acidosis in depolarizing the chromaffin cell and promoting burst firing (Guarina et al. 2017). This is similar to our observation that paxilline enhanced the spiking [Ca^2+^]_i_ response to A1899. It should be noted that TASK‐1 is more sensitive to fall in pH than is TASK‐3 and A1899 is a better inhibitor of TASK1 than TASK3 so it may provide a closer approximation to the effects of acidosis on a mixed population of TASK channels.

In addition to the recently developed TASK channel inhibitors, we also investigated the effects of doxapram. Doxapram is the only respiratory stimulant currently in clinical use. Its effects on ventilation are thought to be primarily due to stimulation of the carotid body (Mitchell and Herbert 1975). It is known to promote neurosecretion from isolated carotid bodies (Anderson Beck et al. 1995) and inhibits heterologously expressed TASK‐1 and TASK‐3 (Cotten et al. 2006). It has also been reported to inhibit BK_Ca_ and K_v_ channels (Peers 1991) but, as discussed above, this alone frequently does not lead to excitation of type‐1 cells. It is therefore hypothesized that the ability of doxapram to act as a respiratory stimulant is due to inhibition of TASK channels (Cotten et al. 2006). The effect of doxapram on native type‐1 cell TASK channels (i.e., heterodimeric TASK 1/3) has not however previously been reported, nor has its effect on [Ca^2+^]_i_ signaling. Our results are therefore the first to confirm that doxapram can indeed inhibit type‐1 cell TASK channels and excite [Ca^2+^]_i_‐signaling. This supports the hypothesis that TASK channel inhibition is key to respiratory stimulation by doxapram. Whilst we cannot at this stage exclude contributory roles for BK_Ca_ and K_V_ inhibition in the context of doxapram, it is clear from the studies discussed above that TASK channel inhibition alone is sufficient to excite type‐1 cells. Moreover selective TASK channel inhibitors (A1899 and PK‐THPP) also act as respiratory stimulants (Cotten 2013). In this respect it is worth noting that the relative potency of these compounds to inhibit TASK channel activity in the type 1 cells (PK‐THPP > A1899 >  doxapram) is the same as that reported for stimulation of ventilation in anesthetized rats (Cotten 2013).

In summary, this study consolidates the hypothesis that the highly expressed background K^+^ channels in type‐1 cells are TASK channels by adding robust pharmacological evidence to existing biophysical and gene knockout evidence. In addition, we confirm that selective inhibition of type‐1 cell TASK channels using these agents was capable of eliciting voltage‐gated Ca^2+^ entry thus supporting the proposed role of these channels in chemosensing. We were further able to confirm a modest role for BK_Ca_ in opposing calcium influx following depolarization induced by TASK inhibition such that the inhibition of these channels by chemostimuli could plausibly also contribute to the calcium response to hypoxia/acidosis. The fact that the [Ca^2+^]_i_ response to hypoxia exceeded that seen to any combination of TASK + BK_Ca_ channel inhibitor, however, suggests that hypoxia probably modulates other channels in addition to TASK and BK_Ca_ and/or modulates [Ca^2+^]_i_ signaling in other ways. Finally our data support the concept that selective TASK channel inhibitors may be of clinical use as respiratory stimulants perhaps to offset the respiratory depression that accompanies the use of a number of general anesthetics and analgesics (Pandit and Buckler 2009; Cotten 2013).

## Conflicts of Interest

The authors have no conflicts of interests to declare.
